# An Unusual Case of Infantile Hepatic Steatosis Caused by Coconut-Based Infant Formula

**DOI:** 10.1097/PG9.0000000000000235

**Published:** 2022-09-30

**Authors:** Paula M. Prieto Jimenez, Esther Jun-Ihn, Michael Matthews, Trang Lollie, Yong Qu, Martin G. Martin

**Affiliations:** From the * Department of Pediatrics at Mattel Children’s Hospital, Pediatric Gastroenterology Fellow at University of California Los Angeles, Los Angeles, CA; † Department of Pediatrics at Mattel Children’s Hospital, Pediatric Hospitalist at University of California Los Angeles, Los Angeles, CA; ‡ Laboratory Assistant at University of California Los Angeles, Los Angeles, CA; § Department of Pathology at Ronal Regan Medical Center, Clinical and Laboratory Pathology Fellow at University of California Los Angeles, Los Angeles, CA; ∥ Biochemical Genetics Laboratory at Kaiser Permanente, Oakland, CA; and; ¶ Department of Pediatrics at Mattel Children’s Hospital, Attending Physician Pediatric Gastroenterology at University of California Los Angeles, Los Angeles, CA.

**Keywords:** coconut oil, fatty liver in neonatal period, hepatic steatosis in newborn, infant hepatomegaly, macrovesicular fatty liver, nonalcoholic fatty liver disease, pediatric hepatomegaly, sea moss

## Abstract

We report a 5-month-old African American male with hepatic steatosis secondary to chronic and exclusive homemade coconut milk formula ingestion. Findings resolved with discontinuation.

## INTRODUCTION

Nonalcoholic fatty liver disease has become the most common liver disease seen in children. Although during infancy, nonalcoholic fatty liver disease most often corresponds to metabolic disorders, in children, it is associated with overweight or obesity. We describe a 5-month-old African American male with hepatic steatosis secondary to chronic and exclusive homemade coconut milk formula ingestion.

## CASE REPORT

A 5-month-old, ex-full-term, unvaccinated, African American infant was referred for elevated transaminase levels and nonbloody, nonbilious emesis since birth. Prior to referral, he was initially diagnosed with gastroesophageal reflux while on breast milk and cows-milk-based formula. He had daily normal-colored stools and was never jaundiced or edematous. Without medical advice, his mother switched him to lactose-sensitive formula at 2 months of age due to persistent emesis and increased fussiness. One month later, she changed him to soy-based formula due to concern for lactose intolerance. At 5 months of age, she switched him to an exclusive homemade formula due to concern for constipation. The recipe consisted of 2 cups of AROY-D coconut milk + 2 cups of water + 1 date + ½ tsp of sea moss powder + 1/4 tsp hemp seeds (Table 1). He was fed 5oz every 4 hours. With this new formula, his fussiness and emesis improved.

**Table 1.** Comparison of nutritional components of commercial coconut milk formula from the case compared with breast milk and Enfamil formula

Commercial Coconut Milk	Coconut milk (100 mL)	Breast milk (100 mL)	Enfamil infant (100 mL)
Carbohydrates	2 gr	7 gr	7.6 gr
Protein	1 gr	1.05 gr	1.35 gr
Fat	14gr	3.9 gr	3.6 gr
Saturated fat	12gr	2 gr	
Cholesterol	0 mg	14 mg	2 mg
Sodium	12 mg	18 mg	18 mg
Potassium	141mg	53 mg	73 mg
Calcium	8 mg	32 mg	53 mg
Iron	0.1 mg	0.03mg	1.2mg
Vitamin D	0 IU	2 IU	51 IU
Calories	204 kcal	66 kcal	66 kcal

Based on the commercial brand of home made formula reported by mother.

**FIGURE 1.**
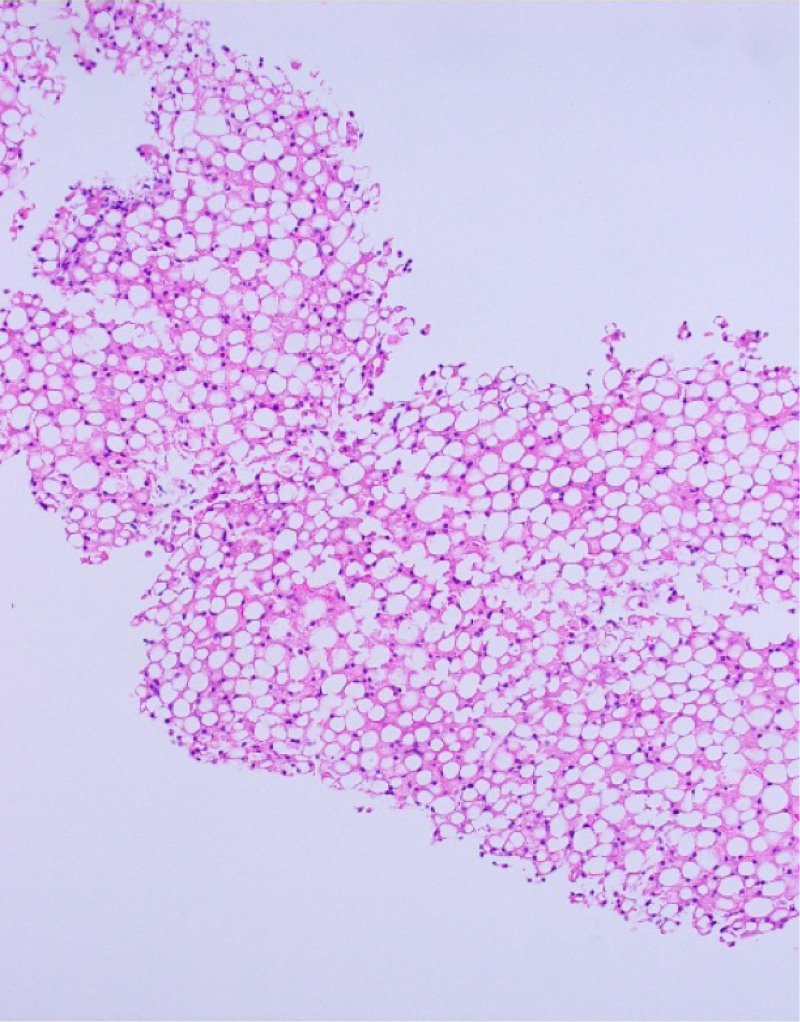
Extensive macrovesicular steatosis, replacing >90% of hepatic parenchyma.

Prenatal history was without complications, with confirmed negative prenatal serologies. Birth weight was 3.8 kg (Z score: 0.86); at presentation, weight was 6.9 kg (Z score: –0.83). No medications were given since birth. Family history was negative for consanguinity, and newborn screen and development were normal. Physical examination during a routine checkup revealed normal cardiac examination but significant hepatomegaly, 4 cm below the right costal margin, without splenomegaly or ascites. Laboratory studies revealed hyponatremia (133 mmol/L), anion gap acidosis (bicarbonate of 16 mmol/L, lactate of 27 mg/dL, and gap of 20 mmol/L), and abnormal liver panel with aspartate aminotransferase 532 U/L, alanine aminotransferase 408 U/L, gamma-glutamyl transferase 235 U/L, and alkaline phosphatase 547 U/L. Total bilirubin (<0.2 mg/dL), complete blood cell count, albumin (4.7 gr/dL), renal function tests, and coagulation studies were normal. Initial abdominal ultrasound showed diffusely echogenic liver parenchyma with hepatomegaly of 10.3 cm (99.7%ile for age). Admission lipid panel was normal except for high triglycerides (174 mg/dL), and fatty acid profile showed elevated lauric and myristic acid but was otherwise normal. 25-OH-Vitamin D was low (10 ng/mL). Further studies including vitamins A, E, and B12, serum iron, and hepatitis panel, cytomegalovirus polymerase chain reaction, Epstein-Barr virus polymerase chain reaction, ceruloplasmin, and autoimmune hepatitis panel were negative or normal. Liver biopsy showed portal tracts without inflammatory infiltrate, but notable extensive macrovesicular steatosis, replacing >90% of the parenchyma without hepatocyte ballooning, Mallory-Denk bodies, microvesicular steatosis, lobular inflammation, cholestasis, or hepatocyte necrosis (Fig. 1). Additional metabolic laboratory tests were abnormal, including plasma amino acids with very low tyrosine and mildly increased threonine, urine organic acids with increased glutaric, 2-hydroxyglutaric, dicarboxylic acids, hexanoylglycine, and suberyglycine, suggestive of possible multiple acyl-CoA dehydrogenase deficiencies, tricarboxylic acid cycle metabolites or mitochondrial insult. Further tests were normal including acylcarnitine profile. In the inpatient setting, he tolerated standard casein hydrolyzed formula with no emesis and regular bowel movements. Elevated transaminase levels were attributed to exclusive homemade coconut formula that the patient had ingested for 1.5 months, and he was discharged with this new formula. On discharge after 5 days of being admitted, his alanine aminotransferase and aspartate aminotransferase improved to 141 U/L and 106 U/L.

One month after the hospitalization, the patient tolerated 5oz of the hydrolyzed formula every 4 hours without symptoms. He weighed 8.0 kg (Z score: 0.11) and had been gaining weight appropriately with an average weight gain of approximately 37g per day. He continued to develop appropriately for age, and his urine organic acid panel, plasma amino acid panel, and liver enzyme tests all normalized. Repeat abdominal ultrasound with elastography showed worsening hepatic steatosis from previous imaging, with persistent hepatomegaly. His shear wave liver stiffness was 5.64 kPa, ruling out compensated chronic advanced liver disease.

The patient continued to follow up every 3 months, and at 14 months old, 9 months after starting the hydrolyzed formula, he was tolerating solid foods and continuing to catch up adequately with respect to developmental milestones and growth (weight 10.7 kg, Z score 0.43). Electrolytes, liver, renal, and lipid panels were normal. Repeat abdominal ultrasound showed improvement of his hepatomegaly with his liver measuring 8.8 cm with a shear wave liver stiffness of 5.02 kPa.

## DISCUSSION

Nonalcoholic fatty liver disease (NAFLD) is the most common chronic liver disease in children.^5^ In infants, inherited metabolic disorders can mimic NAFLD. Among these diseases, medium-chain acyl-coenzyme A dehydrogenase is the most common disorder of fatty acid β-oxidation. It’s typical presentation is between 2 and 3 months of age, with hypoketotic hypoglycemia and vomiting that may progress to lethargy, seizures, and coma triggered by a common illness. These patients can also present with hepatomegaly and liver disease during an acute episode.^7^ Our patient had macrovesicular steatosis instead of microvesicular steatosis as expected in fatty acid oxidation disorders. Furthermore, his emesis was not associated with hypoketotic hypoglycemia, and he presented in the setting of normal mental status as he had no seizures, ruling out metabolic disorders and attributing the changes in the plasma amino acid and urine organic acid to the homemade coconut milk formula.

Our case highlights an unusual etiology of infantile hepatic steatosis. Not only did the coconut milk formula lack essential nutrients for neonates, including carbohydrates, iron, and vitamin D, but it also resulted in the consumption of more than 3 times the standard recommended total fat and saturated fat content per day, which resulted in severe macrovesicular steatosis (Table 1). Whereas coconut water is broadly consumed as a refreshing and nutritious beverage, coconut milk is mainly used in cooking recipes. Coconut milk is the primary source of coconut oil and is the main ingredient in many online homemade formula recipes marketed as vegan and/or natural. No literature documents any benefit of coconut milk in neonates. Coconut oil is a saturated fat that may impair glutathione metabolism and cause oxidative stress leading to NAFLD.^8^

Another additive in the homemade coconut milk formula was sea moss. Sea moss is an alga also known as Irish moss or red seaweed. It is commonly harvested in New England to extract carrageenan, a gelatinous carbohydrate used in baked goods and cosmetics. However, sea moss can also be eaten on its own, and it is often used to thicken soups and stews or as a drink additive. It is considered a prebiotic and tends to accumulate higher concentrations of Na^+^, K^+^, and Zn^+2^ than green seaweeds.^9^ It is known for its role in boosting the immune system, and it’s sometimes sold to help prevent Salmonella infections.

In adults, red seaweed has been shown to increase the effects of anti-hypertensive medications. Although an isolated case of a 40-year-old is reported who had extrahepatic cholestasis with a hepatocellular component due to ingestion of a homemade herbal tea containing Kelp (*Laminaria*)^2^, to our knowledge, no similar cases are reported related to sea moss ingestion in neonates or infants. Also, carrageenan can produce liver injury through sequential activation of natural killer cells and natural killer T cells^10^, which raises our concern of a possible direct hepatotoxic effect of sea moss in our patient. Although herbal supplements are perceived as natural and safe, with the current ongoing expansion of seaweed consumption by the Western population, it is important to investigate the potential adverse effects of herbal supplements and establish specific regulations to prevent their misuse.

By raising awareness of the potential hepatotoxic nature of homemade coconut milk formulas and herbal supplements, we hope to prevent their widespread acceptance until further research can be done.
